# A Novel *FN1* Nucleotide Variant c.3051G>C (p.Trp1017Cys) in a Pediatric Patient with Fibronectin Glomerulopathy: Case Report and Literature Review

**DOI:** 10.3390/jcm15135016

**Published:** 2026-06-27

**Authors:** Lei Sun, Xinyu Kuang, Ying Wu, Wenyan Huang

**Affiliations:** 1 Department of Nephrology and Rheumatology, Shanghai Children’s Hospital, School of Medicine, Shanghai Jiao Tong University, Shanghai 200062, China; sunlei@shchildren.com.cn (L.S.); kuangxy@shchildren.com.cn (X.K.); 2 Department of Pathology, Shanghai Children’s Hospital, School of Medicine, Shanghai Jiao Tong University, Shanghai 200062, China; wuy@shchildren.com.cn

**Keywords:** fibronectin glomerulopathy, *FN1*, pediatrics, next-generation sequencing, diagnosis

## Abstract

**Background/Objectives:** Fibronectin glomerulopathy (FNG) is a rare autosomal dominant inherited kidney disease. Approximately 40% of genetically confirmed FNG cases are associated with likely pathogenic variants in *FN1*. Patients with FNG have similar clinical features as those with chronic nephritis. Due to nonspecific clinical manifestations mimicking common childhood glomerular diseases, FNG poses significant diagnostic challenges in children, frequently resulting in delayed diagnosis. **Case Description:** A 9-year-old Chinese girl presented with manifestations suggestive of acute poststreptococcal glomerulonephritis (APSGN), including edema, hypertension, hypocomplementemia, nephrotic-range proteinuria (3.34 g/24 h), and microscopic hematuria (45–55 cells/HP). Despite resolution of edema and normalized complement C3 after initial therapy, proteinuria and hematuria persisted. Renal biopsy revealed prominent mesangial deposits extending to glomerular capillary walls, with strong fibronectin (FN) immunoreactivity and fibrillary electrondense deposits on electron microscopy. Genetic testing identified a heterozygous *FN1* missense variant c.3051G>C (p.Trp1017Cys) in the proband and her asymptomatic father, classified as likely pathogenic per ACMG guidelines (supporting evidence: PS1, PM2, PP3, PP4). mRNA and cDNA sequencing confirmed the transcription of the mutant allele in the family members. Notably, these transcriptional analyses cannot provide direct evidence for the functional pathogenicity of the variant. The patient received combined angiotensin-converting enzyme inhibitor (ACEI) and angiotensin receptor blocker (ARB) therapy, and renal function remained stable during 3 years of follow-up. **Conclusions:** The *FN1* c.3051G>C represents a novel nucleotide variant, while the corresponding amino acid alteration p.Trp1017Cys has been reported in the previous literature. This case expands the variant spectrum of *FN1* and emphasizes the critical value of renal biopsy and genetic testing for diagnosing FNG in pediatric patients with persistent renal manifestations after suspected APSGN. Family screening is essential for identifying asymptomatic carriers. Our findings also highlight the phenotypic heterogeneity of FNG.

## 1. Introduction

Fibronectin glomerulopathy (FNG) is a rare autosomal dominant glomerular disease characterized by microscopic hematuria, proteinuria, extensive glomerular fibronectin (FN) deposits, and a slowly progressive course leading to end-stage renal disease (ESRD) [1,2]. The underlying pathogenic mechanisms remain incompletely elucidated, and the disease predominantly manifests in individuals aged 10.5 to 30 years [1]. Clinically, FNG mimics chronic glomerulonephritis, presenting with hematuria and proteinuria, and hypertension is observed in approximately 50% of patients.

The *FN1* gene was identified as the causative gene for FNG in 2008 [3]. However, the specific mechanism of *FN1* underlying disease development is still unknown. To date, no standardized treatment regimen has been established for FNG. Due to progressive renal dysfunction, approximately 25% of FNG patients require renal replacement therapy [1,3]. Additionally, only a limited number of FNG pedigrees have been reported in the literature [4]. Herein, we present a case of genetically confirmed FNG in a Chinese child who was initially referred to our hospital with clinical features suggestive of APSGN.

## 2. Case Presentation

A 9-year-old girl was referred to our hospital with clinical manifestations consistent with acute poststreptococcal glomerulonephritis (APSGN), including edema, microscopic hematuria (45–55 cells/HP), nephrotic-range proteinuria (3.34 g/24 h), hypertension (130/88 mmHg), hypocomplementemia (C3: 0.67 g/L), and an antistreptolysin O (ASO) titer at the upper limit of normal (243.00 IU/mL). The patient was initially diagnosed with APSGN and received anti-infective and diuretic therapy. Edema resolved within 1 week, and complement C3 levels normalized (0.91 g/L) after 2 months. However, follow-up evaluations revealed persistent massive proteinuria and microscopic hematuria, prompting the initiation of prednisone therapy (1 mg/kg/day). After 6 months of treatment, glucocorticoid therapy was deemed ineffective, with proteinuria remaining at 0.78 g/24 h and microscopic hematuria at 6–8 cells/HP.

Family urine screening was performed, revealing asymptomatic proteinuria (1.1 g/24 h) in the patient’s father, who had no edema, hypertension, or renal impairment. Given the newly detected proteinuria, the father of the pediatric patient underwent a renal biopsy at another medical institution, with an initial pathological diagnosis of idiopathic nodular glomerulosclerosis. After genetic testing confirmed the familial *FN1* variant, we retrospectively reviewed his original renal pathological slides. No additional fibronectin immunohistochemical staining was performed on the archived biopsy specimens, and his final diagnosis of FNG was revised comprehensively based on genetic results, family pedigree analysis and clinical phenotype.

**Figure 1:**
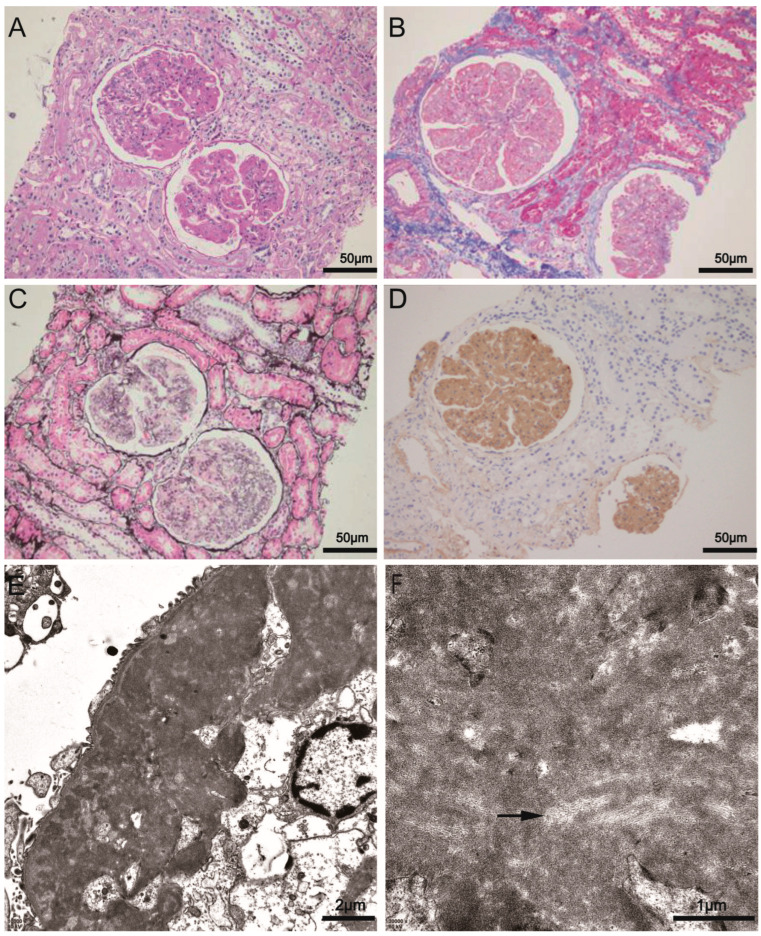
Histopathological findings of the renal biopsy specimen. (**A**). PAS stain: Glomerular enlargement with mesangial expansion; mesangial cell count is not increased. Diffuse PAS-positive staining is evident in glomeruli, consistent with mesangial deposits. Original magnification: ×400; Scale bar: 50 μm. (**B**). Masson stain: Glomeruli show positive red staining with Masson trichrome, indicating ECM accumulation. Original magnification: ×400; Scale bar: 50 μm. (**C**). PAM stain: Glomeruli are negative for periodic acid-methenamine silver staining, a characteristic feature of FNG deposits. Original magnification: ×400; Scale bar: 50 μm. (**D**). Immunohistochemistry for FN: Strong positive staining for FN in glomerular subendothelial and mesangial regions, confirming FN-dominant deposits. Original magnification: ×400; Scale bar: 50 μm. (**E**). Electron microscopy: Extensive electron-dense deposits are visible in the mesangium and subendothelial area. Original magnification: ×10,000; Scale bar: 2 μm. (**F**). Electron microscopy: Fibrillary substructure of deposits with a fibril diameter of 14 nm (arrow), which falls within the 12–16 nm range typical of FNG. Original magnification: ×30,000; Scale bar: 1 μm.

Renal biopsy was performed on the proband. Light microscopy demonstrated prominent mesangial deposits extending to the glomerular capillary walls, with lobular accentuation of strongly PAS-positive and silver-negative material. Minimal proliferative changes were observed in glomeruli, mesangial cells, and intratubular cells. Mild glomerular deposits of immune complexes (IgA, IgG, IgM, and C1q) were detected. Given the typical C3 normalization kinetics of APSGN, these immune complex deposits were interpreted as residual from antecedent streptococcal infection-related immune activation, rather than a primary feature of FNG. Electron microscopy confirmed extensive subendothelial and mesangial electron-dense deposits with a fibrillary substructure (fibril diameter: 14 nm) (Figure 1). Immunohistochemical staining for FN showed strong positivity in the glomerular deposits, confirming prominent FN accumulation in the subendothelial and mesangial regions (Figure 1).

**Figure 2:**
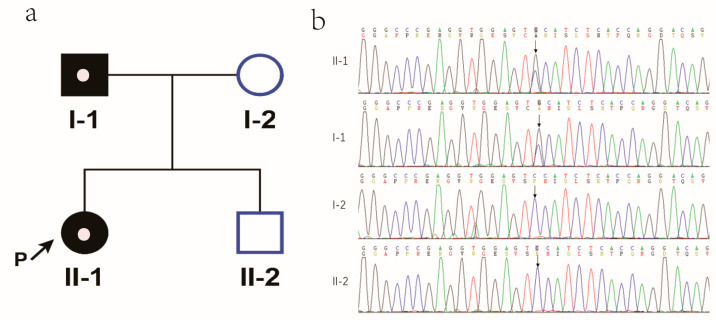
Summary of *FN1* variants detected in DNA. (**a**). Pedigree of the family harboring the *FN1* variant. Filled circles or squares indicate individuals clinically diagnosed with an FNG; unfilled indicates phenotypically normal. The arrow indicates the proband. The white dots indicate *FN1* variant carriers. (**b**). Sanger sequencing chromatogram showing the heterozygous *FN1* c.3051G>C variant (black arrow) in the proband and her father (reverse strand). No variant was detected in the mother.

Genetic testing was performed when FNG was suspected based on pathological findings. Genomic DNA was extracted from peripheral blood samples of the proband and her family members. Whole-exome sequencing (WES) was conducted on the Illumina NovaSeq 6000 platform (Illumina, Inc., San Diego, CA, USA). Standard commercial kits were used for library preparation and target enrichment. Sequence reads were aligned against the human reference genome GRCh37 (hg19). The mean sequencing depth of the *FN1* gene was 100×, and 100% of coding regions achieved coverage above 20×. The bioinformatic pipeline included read quality control, alignment, duplicate removal, variant calling and annotation. Variant filtering criteria were set as follows: (1) Exclude variants with minor allele frequency > 0.01 in public population databases. (2) Retain non-synonymous variants located in functional domains. (3) Prioritize variants consistent with autosomal dominant inheritance pattern. Sanger sequencing was subsequently applied for variant validation in all family members. A heterozygous missense variant in exon 20 of the *FN1* gene, c.3051G>C (p.Trp1017Cys), was identified in both the proband and her father (Figure 2). This variant was absent from public databases (1000 Genomes Project, ExAC, dbSNP, HGMD, and gnomAD v3.1.2) and was predicted to be deleterious by SIFT (score: 0.05), PolyPhen-2 (score: 1.0, probably damaging), and CADD (Phred score: 32). According to the 2015 American College of Medical Genetics and Genomics (ACMG) guidelines, this variant was classified as likely pathogenic, with the following supporting evidence: (1) PS1: A different nucleotide substitution at the identical codon (c.3051G>T) causes the same amino acid change p.Trp1017Cys and has been verified as a likely pathogenic variant for FNG in the published literature; (2) PM2: absent from all major population databases; (3) PP3: multiple in silico tools consistently predict a deleterious effect; (4) PP4: the patient’s phenotype and family history are highly specific for FNG. Specifically, the pathognomonic renal pathological features combined with familial variant co-segregation allow clear differentiation from other overlapping glomerular diseases, which supports the phenotypic specificity required for the PP4 criterion. The father, a carrier of this likely pathogenic variant (LPV), was subsequently re-diagnosed with FNG at the previous institution.

**Figure 3:**
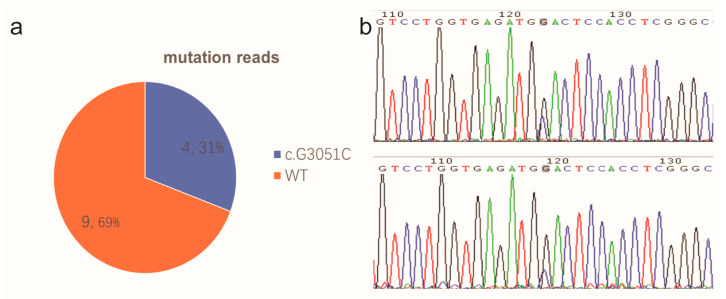
The mRNA analysis of the pedigree. (**a**). RNA-seq reads harboring the *FN1* c.3051G>C variant in the proband’s father, accounting for 31% of total reads (4/13). The low sequencing depth is caused by partial RNA degradation. (**b**). cDNA sequencing chromatograms confirming the heterozygous *FN1* c.3051G>C variant in the proband (upper) and her father (lower), with no variant detected in the mother, consistent with genomic DNA results.

RNA sequencing was performed using an Illumina NovaSeq 6000 platform. Transcription levels were quantified by mapped read counts using HTSeq (version 2.0.5). For the variant locus in the father, the total on-target sequencing depth was 13 reads, among which 4 reads harbored the mutant allele (31% of total reads). The limited read count was attributed to partial RNA degradation during sample transportation and storage. Despite low coverage, the mutant allele was clearly detected, and the allelic ratio is consistent with heterozygous expression. This finding should be interpreted with caution due to the limited sequencing depth. No RNA-seq data for this locus were available for the proband due to severe RNA degradation. To address this limitation, cDNA sequencing was performed on all family members. Sanger sequencing confirmed heterozygosity for the c.3051G>C variant in the proband and her father, with no variant detected in the mother (Figure 3), consistent with genomic DNA results. RNA-seq and cDNA analyses only confirm that the mutant allele can be normally transcribed in vivo. These transcriptional data cannot directly demonstrate the biological function or pathogenic mechanism of this variant. Further functional experiments are required to validate its pathogenicity.

Once the patient was diagnosed with FNG, prednisone therapy was gradually tapered over 3 months. Initial monotherapy with valsartan failed to effectively control persistent proteinuria and hematuria. Considering the progressive nature of FNG and poor response to single-agent renin–angiotensin–aldosterone system (RAAS) inhibition, we initiated combined benazepril (ACEI) and valsartan (ARB) therapy. Although current nephrology guidelines do not recommend routine dual RAAS blockade due to potential risks of hyperkalemia and acute kidney injury, this combination was adopted for this patient after comprehensive assessment of disease activity and individualized risk–benefit analysis. Treatment safety was monitored every 3 months, including measurements of blood pressure, serum potassium, serum creatinine, and 24-h urinary protein excretion. No treatment-related adverse events such as hyperkalemia, hypotension or renal function deterioration were observed. After 3 years of follow-up, the patient’s 24-h urinary protein excretion improved to 0.38 g/24 h, microscopic hematuria persisted (15–25 cells/HP), and renal function remained stable with a normal estimated glomerular filtration rate (eGFR).

## 3. Literature Review

This work is a narrative literature review. A literature search was conducted on 15 January 2025, using the PubMed database. The search term was “fibronectin glomerulopathy”, and the search timeframe was from database inception to 31 December 2024. Inclusion criteria were as follows: (1) Original research articles reporting FNG cases confirmed by renal biopsy and/or genetic testing. (2) Articles containing complete clinical, pathological, and genetic data. Exclusion criteria were as follows: (1) Review articles, editorials, letters, or case reports without original data. (2) Duplicate reports of the same patient. (3) Non-English language articles. As a narrative review, this study did not adopt formal systematic review and meta-analysis protocols, which may lead to potential selection bias. A total of 19 English-language original articles were included. A total of 56 patients (31 males, 25 females) aged 0.2–75 years were included in the analysis. The cohort exhibited a young age distribution, with a mean age of 16.2 years and 15 pediatric cases (<18 years, 26.8%).

Proteinuria and hematuria were the hallmark clinical manifestations of FNG in this cohort. Massive proteinuria (>3.5 g/24 h) was observed in 52.6% (10/19) of patients with available data, followed by moderate proteinuria (1–3.5 g/24 h) in 31.6% (6/19), confirming proteinuria as the dominant initial symptom. Hematuria was detected in 73.1% (19/26) of patients with available data, predominantly microscopic (69.2%, 18/26), with only 1 case of gross hematuria (3.8%, 1/26). Among 34 cases with available blood pressure data, hypertension was present in 47.1% (16/34) of patients. Renal function data were available for 26 cases: 50.0% (13/26) had normal eGFR (≥90 mL/min/1.73 m^2^), 19.2% (5/26) had mild eGFR decline (60–89 mL/min/1.73 m^2^), and 30.8% (8/26) had moderate-to-severe eGFR decline (<60 mL/min/1.73 m^2^) or ESRD.

FNG is an autosomal dominant disease with generally high penetrance, but asymptomatic carriers have been reported in multiple pedigrees, suggesting the presence of incomplete penetrance and variable expressivity. In this cohort, 75.9% (41/54) of patients had a positive family history, while 24.1% (13/54) had no family history of renal disease. This may be attributed to de novo variants, incomplete penetrance, or misdiagnosis/underdiagnosis of affected family members. Missense variants were the predominant genetic defect in FNG, accounting for 85.7% (48/56) of reported cases. The c.2918A>G variant, localized to the III13 repeat domain of *FN1* (a known hot spot for FNG-related variants), was the most common (37.5%). The c.5773T>A and c.5921T>C variants were also frequent, accounting for 14.3% and 16.1%, respectively. Additionally, 2 splice-site single-nucleotide variants and 6 small deletion variants were reported.

The natural course of FNG is characterized by progressive renal function decline, with ESRD being the major long-term outcome. Among 29 cases with prognostic data, 31.0% (9/29) progressed to ESRD requiring dialysis or kidney transplantation, while 58.6% (17/29) maintained stable renal function. Notably, all 9 ESRD patients presented with massive proteinuria at diagnosis, and 4 were pediatric cases, suggesting that severe initial proteinuria (>3.5 g/24 h) and early disease onset (<18 years) are associated with a poor prognosis.

## 4. Discussion

FNG is an autosomal dominant inherited kidney disease caused by likely pathogenic variants in *FN1*, which accounts for approximately 40% of genetically confirmed cases [1,3]. The disease was first described by Assmann et al. in 1995, who identified extensive mesangial and subendothelial deposits with strong fibronectin immunoreactivity in the glomeruli of affected patients [5]. FNG can manifest at any age. Among previously reported cases, the age distribution ranged from 3 to 88 years of age [6]. Clinical features include microscopic hematuria, proteinuria, hypertension, Type IV renal tubular acidosis, and decreased glomerular filtration rate. Most patients who progress to ESRD are between 20 and 60 years of age [5,6]. In the present case, the proband had no known family history of renal disease at presentation. Persistent proteinuria prompted family screening, leading to the identification of asymptomatic proteinuria in her father. To date, both the proband and her father have maintained stable renal function during follow-up, highlighting the variable phenotypic expressivity of FNG even within the same pedigree.

Clinically, proteinuria is the primary clinical feature of FNG. However, FNG exhibits marked phenotypic heterogeneity. Some patients develop proteinuria at an early age. Previous studies have reported that patients with FNG can present with nephrotic syndrome in relatively young patients [7]. Conversely, other patients remain asymptomatic for decades. Asymptomatic carriers and patients with progressive renal injury can coexist within the same pedigree, even when carrying identical likely pathogenic variants [4]. In our case, the proband was initially misdiagnosed with APSGN due to overlapping clinical features, emphasizing the need for differential diagnosis in pediatric patients with persistent renal manifestations.

Given the nonspecific clinical presentation of FNG, a definitive diagnosis relies on renal histopathology. In this case, light microscopy showed enlarged glomeruli with PAS-positive, silver-negative mesangial deposits, consistent with FNG. While FNG is typically characterized by the absence of immune complex deposition [8], previous studies have reported mesangial deposits of IgM and C1q in some cases, with 12 of 19 FNG cases showing co-deposition of fibronectin with immunoglobulins or C3 [9]. The immune complex deposits observed in our patient may be residual from the antecedent APSGN episode, highlighting that concurrent immune complex deposition does not exclude FNG.

Immunohistochemical staining for fibronectin is critical for FNG diagnosis [10]. Previous studies have used monoclonal antibodies (IST-4 and IST-9) to distinguish serum and tissue fibronectin: IST-4 detects both isoforms, while IST-9 is specific for tissue fibronectin. Positive IST-4 staining with weak IST-9 staining suggests predominant serum fibronectin deposition [11]. In our case, strong FN positivity in glomerular deposits confirmed the pathological diagnosis. Electron microscopy findings of 12–16 nm fibrillary substructures in subendothelial and mesangial deposits are also characteristic of FNG [12], further supporting our diagnosis.

Genetic testing confirms FNG diagnosis and facilitates family screening. In 2008, Castelletti et al. first linked FNG to *FN1* mutations, identifying three heterozygous missense variants (p.Y973C, p.W1925R, and p.L1974R) in the heparin-binding domain [3]. Ohtsubo et al. later reported *FN1* mutations in the integrin-binding domain [4]. To date, more than 10 distinct likely pathogenic variants of the *FN1* gene have been identified in patients with FNG. In our study, we identified the variant c.3051G>C (p.Trp1017Cys). It is critical to clarify that the amino acid substitution p.Trp1017Cys has been reported in 2019 via another nucleotide variant c.3051G>T, so the novelty of the present study is limited to the newly discovered nucleotide substitution c.3051G>C, rather than the amino acid alteration [13]. Bioinformatics predictions consistently classified the p.Trp1017Cys amino acid change as functionally detrimental. This variant is located in the HEP-III heparin-binding domain of *FN1*. We hypothesize that this variant disrupts the structural stability of the HEP-III domain, leading to abnormal fibronectin deposition in the glomerulus; this mechanism remains to be validated by functional experiments. Transcriptional analyses in the father confirmed the expression of the mutant allele, but we explicitly acknowledge that such evidence cannot verify the pathogenic function of the variant. Further in vitro and in vivo functional assays are required to explore the exact pathogenic mechanism of this variant.

## 5. Conclusions

This case reports a novel *FN1* nucleotide variant c.3051G>C (p.Trp1017Cys) associated with pediatric FNG. It highlights the importance of clinical vigilance for FNG in children with persistent renal manifestations after suspected APSGN, and the value of an integrated diagnostic approach combining renal biopsy and genetic testing. Family screening is crucial for identifying asymptomatic carriers who may be at risk of future renal disease. Combined ACEI and ARB therapy was administered to the patient after single-agent treatment failure. Regular safety monitoring confirmed no adverse events, and renal function remained stable over 3 years of follow-up. However, the efficacy and safety of dual RAAS blockade in pediatric FNG still need verification in larger prospective cohorts. Further functional studies are needed to elucidate *FN1* variant-specific mechanisms and optimize pediatric FNG management.

## Data Availability

The original contributions presented in this study are included in the article. Further inquiries can be directed to the corresponding author.
